# Physician Awareness of Chagas Disease, USA

**DOI:** 10.3201/eid1605.091440

**Published:** 2010-05

**Authors:** Kelly K. Stimpert, Susan P. Montgomery

**Affiliations:** Atlanta Research and Education Foundation, Atlanta, GA, USA (K.K. Stimpert); Centers for Disease Control and Prevention, Atlanta (K.K. Stimpert, S.P. Montgomery)

**Keywords:** Chagas disease, Trypanosoma cruzi, Hispanics, Hispanic Americans, pilot projects, parasitic diseases, parasites, qualitative research, minority health, letter, *Suggested citation for this article:* Stimpert KK, Montgomery SP. Physician awareness of Chagas disease, USA [letter]. Emerg Infect Dis [serial on the Internet]. 2010 May [*date cited*]. http://www.cdc.gov/EID/content/16/5/871.htm

**To the Editor**: The year 2009 was the 100th anniversary of Carlos Chagas’s discovery of the disease caused by the parasite *Trypanosoma cruzi*, now known as Chagas disease. Spread by infected bugs called triatomines, the disease is endemic throughout much of Mexico and Central and South America. An estimated 8–11 million persons in Latin America have the disease (*1*). *T. cruzi* infection causes more public health problems with long-term consequences in Latin America than any other parasitic disease (*2*).

Although earlier estimates suggested that 100,000 infected persons may live in the United States (*3*), recent data suggest that ≈300,000 persons are infected (*4*). Most of these persons are unaware that they are infected. Antiparasitic drugs to treat Chagas disease have not been approved by the US Food and Drug Administration and are only available in the United States through the Centers for Disease Control and Prevention (CDC) for use under investigational protocols for compassionate treatment.

Screening of the US blood supply for Chagas disease began in early 2007; more than 500 donors with *T. cruzi* infection were identified within the first 18 months (*5*). However, to date, only ≈11% of *T. cruzi*–positive donors (or their physicians) have contacted CDC for treatment consultations (CDC, unpub. data). One possible explanation is the limited awareness of Chagas disease in the United States among health professionals, the general public, and even among Mexican-born Americans (K.K. Stimpert and S.P. Montgomery, unpub. data).

To assess healthcare provider awareness of Chagas disease, a study was conducted by MedscapeCME (www.medscape.com) with technical support from CDC. MedscapeCME, a leading provider of online clinical and medical information and continuing medical education (CME) for physicians and other healthcare professionals, carried out a knowledge, attitudes, and practices (KAP) assessment of physician members of its website. Researchers anticipated that the results of the study would inform larger formal studies among healthcare providers in geographic areas where population demographics and blood donor screening results suggest that persons are at risk for Chagas disease.

The KAP study was based on five 10-question surveys, which were posted on the MedscapeCME website. Each survey was tailored for a specific specialty: primary care, infectious disease, cardiology, obstetrics/gynecology, and transplantation medicine; they were designed to measure basic knowledge of Chagas disease epidemiology and consideration of Chagas disease risk among these physicians.

MedscapeCME members were given the option of participating in the surveys when they logged onto each specialty site. MedscapeCME membership requirements include online registration, which is validated by confirming American Medical Association membership. Each member could complete the survey only once. All surveys were launched on December 11, 2008, but discontinued at different times (median 39 days).

Although familiarity with Chagas disease varied by specialty, the results suggested that a general lack of awareness was common across all groups (Table). This awareness deficit was most pronounced in obstetricians and gynecologists and least pronounced in infectious disease physicians (Table). The 7 responses to questions outlined in the Table may be placed in 3 categories: 1) general awareness of Chagas disease (questions 1 and 3), 2) confidence in Chagas disease knowledge and consideration of risk (questions 2 and 5), and 3) clinical aspects of Chagas disease (questions 4, 6, and 7). Across all 3 domains, obstetricians and gynecologists reported low knowledge of Chagas disease and a low level of confidence in their knowledge. Infectious disease physicians had the highest levels of knowledge and confidence across all domains, except for responses to question 1 (Table).

**Table:** Reponses to MedscapeCME knowledge, attitudes, and practices survey questions reflecting lack of knowledge about Chagas disease, by specialty*

Response	No. (%) respondents†				
	Cardiology, n = 280	Infectious disease, n = 167	OB/GYN, n = 292	Primary care, n = 278	Transplantation, n = 125
Never heard of Chagas disease‡	63 (23)	31 (19)	138 (47)	38 (14)	35 (25)
Not at all confident of Chagas disease knowledge being up to date§	87 (44)	31 (27)	86 (68)	101 (47)	41 (48)
Did not know parasite causes Chagas disease¶	21 (16)	6 (5)	42 (33)	35 (16)	15 (17)
Did not know cardiac and/or gastrointestinal disease are manifestations of Chagas disease#	15 (8)	10 (9)	38 (30)	24 (11)	11 (13)
Never considers risk for Chagas disease in patients**	51 (34)	30 (29)	66 (60)	83 (43)	29 (39)
Did not know in what percentage of patients with chronic infection clinical disease develops††	66 (37)	30 (28)	60 (56)	93 (48)	35 (47)
Did not know Chagas disease symptoms‡‡	41 (23)	15 (14)	53 (48)	52 (27)	22 (29)

*OB/GYN, obstetrics/gynecology. †Percentages are calculated based on number of respondents per question, by specialty. ‡Question 1: Have you heard of Chagas disease? Answer choices: yes, no. §Question 2: How confident are you that your Chagas disease knowledge is up to date? Answer choices: very confident, confident, somewhat confident, not at all confident. ¶Question 3: Chagas disease is caused by a ___? Answer choices: bacterium, virus, parasite, fungus, I don’t know. #Question 4: People with chronic Chagas disease may have (check all that apply)? Answer choices: cardiac conduction abnormalities, cardiomyopathy, megacolon, co-clinical manifestations, I don’t know. **Question 5: How often do you consider the risk for Chagas disease in your patient population? Answer choices: never, rarely, sometimes, frequently, always. ††Question 6: Approximately what percentage of patients with chronic Chagas infection eventually develop clinical disease? Answer choices: <20%, 21%–40%, >40%, I don’t know. ‡‡Question 7: Chagas disease symptoms are? Answer choices: Acute for several weeks then immediately symptomatic, Acute for several weeks asymptomatic for years to decades then sometimes symptomatic, There are no symptoms of Chagas disease, I don’t know.

Survey results suggest substantial knowledge deficits among physicians, especially among obstetricians and gynecologists. The apparent lack of knowledge in the obstetrics and gynecology community is of particular concern because Chagas disease can be transmitted congenitally (*6*). Because Chagas disease may also be transmitted by organ transplantation, the proportion of respondents from this specialty who indicated they never consider risk for Chagas disease in their patients (39%) is also notable. In fact, many physicians surveyed never consider the risk for Chagas disease in their patient population (29%–60%) and are not at all confident that their knowledge of Chagas disease is current (27%–68%).

The study has some limitations. The conclusions are drawn from a study in which convenience samples from selected populations were used and are thus not generalizable to larger healthcare provider populations in the United States. The high participation rate of transplant surgeons may reflect a fluctuation in membership numbers. In addition, transplant surgeons may have an increased interest in Chagas disease because of recent instances of transplant-associated transmission (*7**,**8*).

These preliminary data suggest a substantial knowledge deficit regarding Chagas disease among healthcare providers, which could have a negative effect on patient health if Chagas disease is not recognized and appropriately treated. CDC plans to conduct larger scale KAP surveys of physicians in areas where blood donor screening results suggest relatively high prevalence in the underlying communities. For example, *T. cruzi* infection prevalence in blood donors in Florida and California was reported to be 1/3,700 and 1/8,300, respectively (*5*). Future studies will help document why Chagas disease is underrecognized in the United States and further demonstrate the need for educating healthcare providers about this disease.
